# Pre- and Post-diagnosis Diabetes as a Risk Factor for All-Cause and Cancer-Specific Mortality in Breast, Prostate, and Colorectal Cancer Survivors: a Prospective Cohort Study

**DOI:** 10.3389/fendo.2020.00060

**Published:** 2020-02-18

**Authors:** Huan Tao, Adrienne O'Neil, Yunseon Choi, Wei Wang, Junfeng Wang, Yafeng Wang, Yongqian Jia, Xiong Chen

**Affiliations:** ^1^Department of Hematology and Research Laboratory of Hematology, West China Hospital, Sichuan University, Chengdu, China; ^2^The Centre for Innovation in Mental and Physical Health and Clinical Treatment, Deakin University, Geelong, VIC, Australia; ^3^Melbourne School of Population and Global Health, University of Melbourne, Carlton, VIC, Australia; ^4^Department of Radiation Oncology, Busan Paik Hospital, Inje University College of Medicine, Busan, South Korea; ^5^School of Mathematical Sciences, Shanghai Jiao Tong University, Shanghai, China; ^6^Julius Center for Health Sciences and Primary Care, University Medical Center Utrecht, Utrecht University, Utrecht, Netherlands; ^7^Department of Epidemiology and Biostatistics, School of Health Sciences, Wuhan University, Wuhan, China; ^8^Department of Endocrinology, The First Affiliated Hospital of Wenzhou Medical University, Wenzhou, China

**Keywords:** diabetes, all-cause, cancer, cardiovascular disease, mortality, cohort study

## Abstract

**Objective:** The relationship between diabetes and all- and cause-specific mortality in individuals with common cancers (breast, colorectal, and prostate) remains both under-researched and poorly understood.

**Methods:** Cancer survivors (*N* = 37,993) from the National Health Interview Survey with linked data retrieved from the National Death Index served as our study participants. Cox proportional-hazards models were used to assess associations between pre- and post-diabetes and all-cause and cause-specific mortality.

**Results:** Over a median follow-up period of 13 years, 2,350 all-cause, 698 cancer, and 506 CVD deaths occurred. Among all cancer survivors, patients with diabetes had greater risk of: all-cause mortality [hazard ratio (HR) 1.35, 95% CI = 1.27–1.43], cancer-specific mortality (HR: 1.14, 95% CI = 1.03–1.27), CVD mortality (HR: 1.36, 95% CI = 1.18–1.55), diabetes related mortality (HR: 17.18, 95% CI = 11.51–25.64), and kidney disease mortality (HR: 2.51, 95% CI = 1.65–3.82), compared with individuals without diabetes. The risk of all-cause mortality was also higher amongst those with diabetes and specific types of cancer: breast cancer (HR: 1.28, 95% CI = 1.12–1.48), prostate cancer (HR: 1.20, 95% CI = 1.03–1.39), and colorectal cancer (HR: 1.29, 95% CI = 1.10–1.50). Diabetes increased the risk of cancer-specific mortality among colorectal cancer survivors (HR: 1.36, 95% CI = 1.04–1.78) compared to those without diabetes. Diabetes was associated with higher risk of diabetes-related mortality when compared to non-diabetic breast (HR: 9.20, 95% CI = 3.60–23.53), prostate (HR: 18.36, 95% CI = 6.01–56.11), and colorectal cancer survivors (HR: 12.18, 95% CI = 4.17–35.58). Both pre- and post-diagnosis diabetes increased the risk of all-cause mortality among all cancer survivors. Cancer survivors with diabetes had similar risk of all-cause and CVD mortality during the second 5 years of diabetes and above 10 years of diabetes as compared to non-diabetic patients.

**Conclusions:** Diabetes increased the risk of all-cause mortality among breast, prostate, and colorectal cancer survivors, not for pre- or post-diagnosis diabetes. Greater attention on diabetes management is warranted in cancer survivors with diabetes.

## Introduction

Diabetes is a major public health burden. The prevalence of diabetes is projected to increase to 439 million adults by 2030. By this time, the disease burden in adults living with diabetes is estimated to increase by 20 and 69% in developing and developed countries, respectively (1, 2).

Diabetes mellitus (DM) and cancer are two common diseases affecting aging populations worldwide, which commonly co-occur. These associations are complex and may be cancer specific (3). On one hand, strong evidence links diabetes to an increased risk of breast and colorectal cancer onset (4, 5). On the other hand, there is some evidence it may be associated with decreased risk of prostate cancer onset (6). For those with a cancer diagnosis or history of the condition, there is evidence that diabetes may increase overall mortality risk. Previous studies have reported that diabetes increased the all-cause and cancer-specific mortality for cancer survivors compared to those without diabetes (7), including breast (8), colorectal (7–9), and prostate cancer survivors (10, 11). Interestingly, individuals with both diabetes and a history of cancer also have higher rates of cardiovascular mortality when compared to those without diabetes among the general population (12, 13). However, the relationship between diabetes and all- and cause-specific mortality in individuals with common cancers (breast, colorectal, and prostate) remains both under-researched and poorly understood.

This study aimed to estimate the associations of pre- and post-diagnosis diabetes, and duration of diabetes with all-cause, cancer-specific and CVD mortality among U.S. breast, prostate and colorectal cancer survivors in a large prospective cohort study.

## Methods

### Study Population

The National Health Interview Survey (NHIS) is a stratified, multistage probability survey that samples an average of 57,000 adults per year to estimate the health of the U.S. population, the prevalence and incidence of disease, the extent of disability, and the use of health care services. One adult is randomly selected from each selected household for a detailed interview on health and other behaviors.

**Figure 1:**
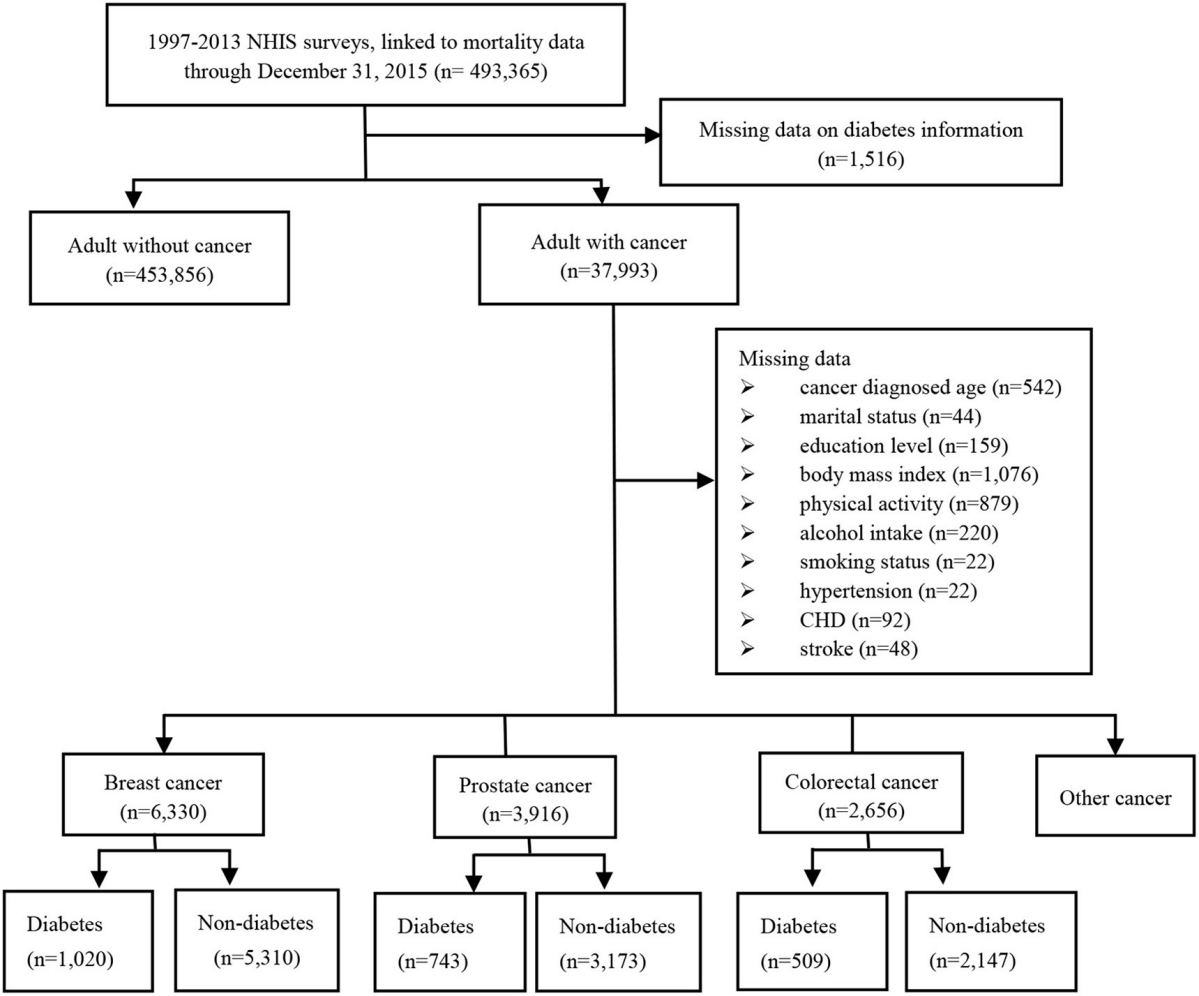
Analytic sample selection.

A total of 493,365 adults from the 13 cross-sectional waves (i.e., from 1997 to 2013) and their linked mortality data ending in December 31, 2015 was included in this analysis. After excluding missing data on diabetes, we identified 37,993 adults cancer survivors, including 6,330 breast cancer patients, 3,916 prostate cancer patients, and 2,656 colorectal cancer patients. A total of 5,174 cancer survivors diagnosed as diabetes, including 1,020 in breast cancer patients, 743 in prostate cancer patients and 509 in colorectal cancer patients. Totally, we identified 4,724 patients with diabetes prior to cancer diagnosis and 450 patients with diabetes after cancer diagnosis (Figure 1).

### Diabetes

Self-reported diabetes was based on responses to questions “Have you ever been told by a doctor or other health professional that you had…” Women reporting diabetes occurring only during pregnancy, or adults who responded they had borderline diabetes were considered to not have diabetes. Diabetes was dichotomized into the presence or absence of diabetes (14, 15). Duration of diabetes was calculated by the present age minus the age at diagnosis of diabetes, and was categorized as <5, 5–10, and >10 years. After excluding individuals without diabetes and those with possible type 1 diabetes adults defined by using insulin and age of onset <30 years old which has been validated as accurate in 97% of cases (16).

### Covariables

We included demographic variables (i.e., race, gender, education, and marital status), lifestyle variables (i.e., body mass index, physical activity, and alcohol consumption) and comorbid conditions [i.e., hypertension, coronary heart disease (CHD), and stroke]. Body mass index (BMI) was calculated as weight in kilograms divided by height squared (<25, 25–30, and >30 kg/m^2^). A participant's physical activity was categorized into three groups according to metabolic equivalent task (MET) hours per week (low, 600 MET-h/wk; moderate, 600–3,000 MET-h/wk; high, >3,000 MET-h/wk). Alcohol consumption was categorized into three groups: lifetime abstainer; former drinker; current drinker. Cancer was defined as the presence of the same National Cancer Registry with a specific code (C code) more than three times within a year or an in patient hospitalization with a C code (17). Cancer diagnosed age was defined as the age at diagnosis of cancer and was categorized into <45, 45–65, and >65 years. Duration of cancer was calculated by the present age minus the cancer diagnosed age.

### Mortality

Ascertainment of mortality was established using the International Classification of Diseases-10th Revision codes, and study outcomes were defined using as follows: (1) all-cause mortality; (2) cancer-specific mortality (i.e., codes C00–C97); (3) CVD-mortality (i.e., codes I00–I09, I11, I13, and I20–I51, I60–I69); (4) diabetes mellitus (E10–E14); (5) chronic lower respiratory diseases (J40–J47); (6) influenza and pneumonia (J09–J18); (7) Alzheimer disease (G30) and (8) nephritis, Nephrotic syndrome and nephrosis (N00–N07, N17–N19, N25–N27).

### Statistical Analysis

We compared participants with and without diabetes for basic characteristics using chi-square test to examine categorical differences in the weighted percentages. According to the baseline hazard to capture the increase in hazard due to aging, age was used as the underlying timescale. To evaluate the association of diabetes with all-cause, cancer and CVD mortality, we computed multivariable-adjusted hazard ratios (HRs) and 95% confidence intervals (CI) using cause-specific Cox proportional hazards models. All analyses were adjusted for age (using it as a timescale), sex, race, education level, income level, marital status, body mass index, smoking status, physical activity, alcohol intake, cancer diagnosed age, duration of cancer, history of hypertension, CHD, stroke, and missing data. We also analyzed the association between duration of diabetes and the risk of disease-specific and all-cause mortality. Besides, the relationship between pre- and post-diagnosis diabetes and mortality was examined among the 5,174 men and women cancer survivors. Two sensitivity analyses were also performed: (1) exclusion of individuals who died within the first 2 years; (2) exclusion of participants with CHD or stroke at interview; (3) excluding individuals with possible type 1 diabetes (defined by being on insulin and having an age of onset <30 years).

All analyses incorporated the complex survey design and were performed by using STATA version 15.0 (Stata Corp, College Station, TX, USA). Two-sided *p*-values <0.05 were considered significant for statistical inferences.

## Results

Descriptive statistics were reported in Table 1. The mean age of cancer survivors was 63 years at baseline. Compared to cancer survivors without diabetes, cancer survivors with diabetes were more likely to be obesity (i.e., BMI > 30 kg/m^2^) and be less physically active (i.e., low physical activity). Cancer survivors with diabetes tend to have a degree less than high school (24.1% in patients with diabetes, 15.09% in patients without diabetes). Patients with diabetes tend to have a history of hypertension (75.51% in patients with diabetes, 43.49% in patients without diabetes), CHD (24.17% in patients with diabetes, 9.52% in patients without diabetes), and stroke (12.44% in patients with diabetes, 5.38% in patients without diabetes).

**Table 1:** Basic characteristics.

**Characteristics**	**Subgroups**	**Adult with diabetes (*n* = 5,174)**	**Adults without diabetes (*n* = 29,715)**	***P*-value**
		***N* (%)**	***N* (%)**	
Age				
	18–45 years	260 (5.08%)	5,059 (16.71%)	<0.001
	45–65 years	1,874 (35.91%)	11,424 (39.12%)	
	≥65 years	3,409 (59.01%)	15,425 (44.17%)	
Sex				<0.001
	Male	2,476 (49.13%)	12,036 (41.25%)	
	Female	3,067 (50.87%)	19,872 (58.75%)	
Race				<0.001
	Hispanic	500 (6.59%)	1,851 (3.93%)	
	Non-Hispanic White	4,143 (81.96%)	27,006 (88.99%)	
	Non-Hispanic Black	764 (9.22%)	2,363 (5.07%)	
	Non-Hispanic Other	136 (2.23%)	688 (2.01%)	
Education level				<0.001
	Less than high school degree	1,483 (24.10%)	5,531 (15.09%)	
	High school degree	1,717 (32.46%)	9,054 (28.75%)	
	More than high school degree	2,315 (43.44%)	17,187 (56.16%)	
Income level				<0.001
	Low	903 (12.02%)	3,796 (8.81%)	
	Moderate	3,306 (59.37%)	16,748 (49.84%)	
	High	1,334 (28.61%)	11,364 (41.35%)	
Marital status				<0.001
	Married/Living with partner	2,611 (61.74%)	16,273 (65.70%)	
	Widowed/Divorced/Separated	2,547 (32.83%)	12,804 (27.69%)	
	Never married	381 (5.43%)	2,791 (6.61%)	
Body mass index				<0.001
	<25 (kg/m^2^)	1,078 (19.95%)	12,927 (41.04%)	
	25–30 (kg/m^2^)	1,827 (33.88%)	11,161 (36.53%)	
	>30 (kg/m^2^)	2,473 (46.17%)	6,885 (22.43%)	
Physical activity				<0.001
	Low (<600 MET-h/wk)	4,069 (73.19%)	19,331 (60.14%)	
	Moderate (600–3,000 MET-h/wk)	1,100 (22.05%)	9,107 (31.41%)	
	High (>3,000 MET-h/wk)	237 (4.76%)	2,462 (8.45%)	
Smoking status				<0.001
	Never smoking	2,424 (43.02%)	14,416 (45.28%)	
	Former smoking	2,382 (44.37%)	11,384 (36.75%)	
	Current smoking	700 (12.61%)	5,900 (17.96%)	
Alcohol intake				<0.001
	Lifetime abstainer	1,450 (25.17%)	6,224 (18.09%)	
	Former drinker	1,945 (34.76%)	7,100 (21.63%)	
	Current drinker	2,066 (40.07%)	18,129 (60.27%)	
Hypertension				<0.001
	Yes	4,243 (75.51%)	14,498 (43.49%)	
	No	1,297 (24.49%)	17,383 (56.51%)	
Stroke				<0.001
	Yes	707 (12.44%)	1,849 (5.38%)	
	No	4,826 (87.56%)	30,014 (94.62%)	
Coronary heart disease				<0.001
	Yes	1,270 (24.17%)	3,090 (9.52%)	
	No	4,241 (75.83%)	28,740 (90.48%)	
Cancer diagnosed age				<0.001
	<45	1,295 (24.04%)	11,599 (37.79%)	
	45–65	2,540 (46.63%)	12,537 (40.05%)	
	>65	1,708 (29.33%)	7,772 (22.15%)	

**Figure 2:**
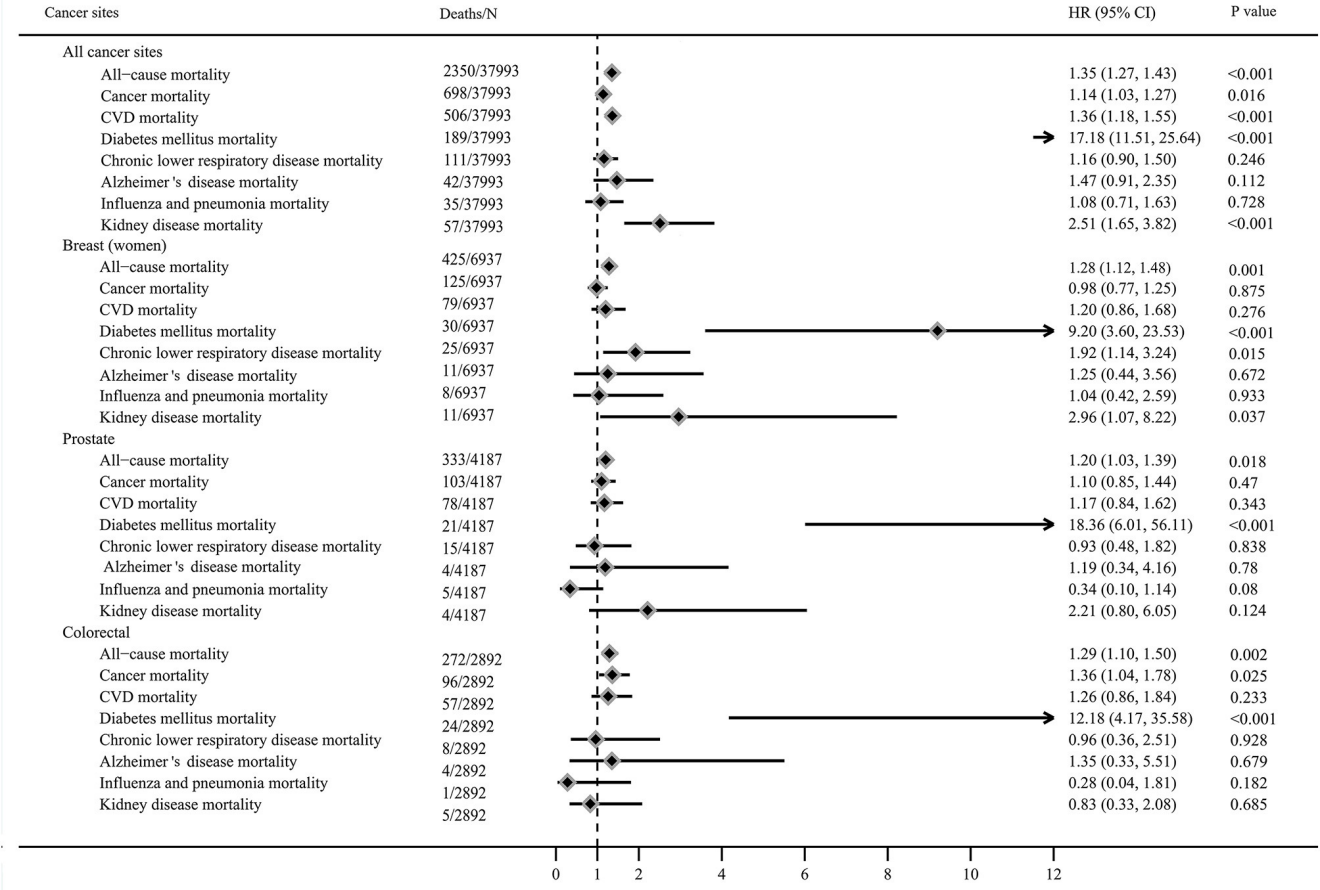
Mortality of cancer survivors with diabetes compared to non-diabetic cancer survivors (adjusted for age, sex, race, education level, income level, marital status, body mass index, smoking status, alcohol intake, physical activity, cancer diagnosed age, duration of cancer, history of hypertension, coronary heart disease and stroke, and missing data).

After a median follow-up period of 13 years, 2,346 all-cause, 698 cancer-specific, and 506 CVD deaths occurred. Figure 2 showed the risk of all-cause, cancer and CVD mortality among diabetic cancer survivors compared to non-diabetic cancer survivors. Compared to non-diabetes cancer survivors, cancer survivors with diabetes had a 35% higher risk of all-cause mortality (HR: 1.35, 95% CI = 1.27–1.43), 14% higher risk of cancer mortality (HR: 1.14, 95% CI = 1.03–1.27), and 36% higher risk of CVD mortality (HR: 1.36, 95% CI = 1.18–1.55). These relationships were stronger for diabetes related mortality (HR: 17.18, 95% CI = 11.51–25.64) and kidney disease mortality 2.51 for (95% CI = 1.65–3.82) who were diabetic survivors when compared to non-diabetic survivors, but no associations were observed between diabetes and risk of chronic lower respiratory disease, influenza and pneumonia, and Alzheimer disease mortality.

The HRs for all-cause mortality varied from 1.20 to 1.30 among survivors with diabetes diagnosed with breast cancer (HR: 1.28, 95% CI = 1.12–1.48), prostate cancer (HR: 1.20, 95% CI = 1.03–1.39), and colorectal cancer (HR = 1.29, 95% CI = 1.10–1.50). Diabetes increased the risk of cancer-specific mortality among colorectal cancer survivors (HR: 1.36, 95% CI = 1.04–1.78) compared to non-diabetic survivors, but not in breast and prostate cancer survivors. Diabetes was associated with higher risk of diabetes related mortality in breast (HR: 9.20, 95% CI = 3.60–23.53), prostate (HR: 18.36, 95% CI = 6.01–56.11), and colorectal cancer survivors (HR: 12.18, 95% CI = 4.17–35.58), compared to non-diabetic cancer survivors. Diabetes increased the risk of chronic lower respiratory disease mortality (HR: 1.92, 95% CI = 1.14–3.24) and kidney disease mortality (HR: 2.96, 95% CI = 1.07–8.22) among breast survivors compared to non-diabetic survivors, but not in colorectal and prostate cancer survivors.

Pre-diagnosis diabetes increased the risk of all-cause mortality (HR: 1.35, 95% CI = 1.27–1.44), cancer mortality (HR: 1.14, 95% CI = 1.02–1.28), CVD mortality (HR: 1.38, 95% CI = 1.20–1.59), diabetes mellitus mortality (HR: 17.82, 95% CI = 11.86–26.79), Alzheimer's disease mortality (HR: 1.66, 95% CI = 1.01–2.72), and kidney disease mortality (HR: 2.45, 95% CI = 1.60–3.75), respectively among all cancer survivors. Post-diagnosis diabetes increased the risk of all-cause mortality (HR: 1.35, 95% CI = 1.12–1.63) and diabetes mellitus mortality (HR: 8.62, 95% CI = 3.83–19.39), but not in other mortality. For specific cancer sites, pre-diagnosis diabetes increased the risk of all-cause mortality in prostate (HR: 1.21, 95% CI = 1.01–1.43) and breast cancer survivors (HR: 1.31, 95% CI = 1.10–1.57), not in colorectal cancer survivors. Post-diagnosis diabetes did not confer obvious increased risk of all-cause mortality for prostate and colorectal cancer sites except breast cancer (HR: 1.25, 95% CI = 1.02–1.52). Additional information were detailed in Table 2.

**Table 2:** Mortality of cancer survivors with pre-diagnosis diabetes group compared to non-diabetic group.

**Cancer sites**	**Mortality**	**Deaths**	**Adults without diabetes (*n* = 29,715)**	**Adults with diabetes (*****n*** **=** **5,174)**			
				**Pre-diagnosis diabetes** **(*****n*** **=** **4,724)**		**Post-diagnosis diabetes** **(*****n*** **=** **450)**	
			**HR**	**HR (95% CI)**	***P*-value**	**HR (95% CI)**	***P*-value**
**All cancer sites**							
	All-cause mortality	2,350	1.00 (refer)	1.35 (1.27–1.44)	<0.001	1.35 (1.12–1.63)	0.002
	Cancer mortality	698	1.00 (refer)	1.14 (1.02–1.28)	0.02	1.23 (0.85–1.76)	0.267
	CVD mortality	506	1.00 (refer)	1.38 (1.20–1.59)	<0.001	1.07 (0.67–1.70)	0.771
	Chronic lower respiratory disease mortality	111	1.00 (refer)	1.12 (0.85–1.46)	0.429	1.73 (0.84–3.59)	0.138
	Diabetes mellitus mortality	189	1.00 (refer)	17.82 (11.86–26.79)	<0.001	8.62 (3.83–19.39)	<0.001
	Alzheimer's disease mortality	42	1.00 (refer)	1.66 (1.01–2.72)	0.044	0.37 (0.09–1.60)	0.185
	Influenza and pneumonia mortality	35	1.00 (refer)	0.98 (0.61–1.55)	0.917	2.18 (0.68–6.96)	0.188
	Kidney disease mortality	57	1.00 (refer)	2.45 (1.60–3.75)	<0.001	1.92 (0.66–5.61)	0.234
**Breast (women)**	All-cause mortality	425	1.00 (refer)	1.31 (1.10–1.57)	0.003	1.25 (1.02–1.52)	0.03
	Cancer mortality	125	1.00 (refer)	NA	NA	NA	NA
	CVD mortality	79	1.00 (refer)	2.05 (0.51–8.24)	0.313	NA	NA
	Diabetes mellitus mortality	30	1.00 (refer)	NA	NA	NA	NA
	Chronic lower respiratory disease mortality	25	1.00 (refer)	NA	NA	14.83 (3.67–59.88)	<0.001
	Alzheimer's disease mortality	11	1.00 (refer)	NA	NA	NA	NA
	Influenza and pneumonia mortality	8	1.00 (refer)	NA	NA	NA	NA
	Kidney disease mortality	11	1.00 (refer)	79.35 (4.93–1,277.73)	0.002	NA	NA
**Prostate**	All-cause mortality	333	1.00 (refer)	1.21 (1.01–1.43)	0.036	1.18 (0.93–1.51)	0.18
	Cancer mortality	103	1.00 (refer)	1.38 (0.38–4.98)	0.621	0.69 (0.07–6.71)	0.75
	CVD mortality	78	1.00 (refer)	NA	NA	NA	NA
	Diabetes mellitus mortality	21	1.00 (refer)	NA	NA	NA	NA
	Chronic lower respiratory disease mortality	15	1.00 (refer)	3.62 (1.12–11.72)	0.032	NA	NA
	Alzheimer's disease mortality	4	1.00 (refer)	16.44 (1.86–145.26)	0.012	NA	NA
	Influenza and pneumonia mortality	5	1.00 (refer)	NA	NA	NA	NA
	Kidney disease mortality	4	1.00 (refer)	NA	NA	NA	NA
**Colorectal**	All-cause mortality	272	1.00 (refer)	1.19 (0.77–1.84)	0.441	0.98 (0.33–2.93)	0.969
	Cancer mortality	96	1.00 (refer)	1.89 (0.51–7.02)	0.34	NA	NA
	CVD mortality	57	1.00 (refer)	NA	NA	NA	NA
	Diabetes mellitus mortality	24	1.00 (refer)	NA	NA	NA	NA
	Chronic lower respiratory disease mortality	8	1.00 (refer)	NA	NA	NA	NA
	Alzheimer's disease mortality	4	1.00 (refer)	NA	NA	NA	NA
	Influenza and pneumonia mortality	1	1.00 (refer)	NA	NA	NA	NA
	Kidney disease mortality	5	1.00 (refer)	NA	NA	NA	NA

*CVD, cardiovascular disease; HR, hazard ratios*.

*Adjusted for age, sex, race, education level, income level, marital status, body mass index, smoking status, alcohol intake, physical activity, cancer diagnosed age, duration of cancer, history of hypertension, coronary heart disease and stroke, and missing data*.

As summarized in Table 3, cancer survivors with diabetes had similar risk of all-cause and CVD mortality during the second 5 years of diabetes [(HR: 1.44, 95% CI = 1.29–1.60); (HR: 1.43, 95% CI = 1.13–1.82)] and above 10 years [(HR = 1.41, 95% CI = 1.30–1.52); (HR: 1.45, 95% CI = 1.22–1.71)] as compared to non-diabetic participants. However, cancer survivors with duration of diabetes 5–10 years (HR: 1.40, 95% CI = 1.15–1.70) achieved the highest risk of cancer mortality compared to those with duration of diabetes <5 years or more than 10 years. Compared to non-diabetic survivors, cancer survivors with diabetes with duration for 0–5 years (HR: 10.97, 95% CI = 6.28–19.16), 5–10 years (HR: 11.04, 95% CI = 6.20–19.64), and more than 10 years (HR: 24.11, 95% CI = 15.55–37.38) were at increased risk of diabetes related mortality.

**Table 3:** Duration of diabetes among cancer survivors compared to those without diabetes.

**Cancer sites**	**Mortality**	**Adults without diabetes**	**Adults with diabetes**					
			**0**–**5 years**		**5**–**10 years**		**>10 years**	
		**HR**	**HR (95% CI)**	***P*-value**	**HR (95% CI)**	***P*-value**	**HR (95% CI)**	***P*-value**
**All cancer sites**								
	All-cause mortality	1.00 (refer.)	1.21 (1.09, 1.33)	<0.001	1.44 (1.29–1.60)	<0.001	1.41 (1.30–1.52)	<0.001
	Cancer mortality	1.00 (refer.)	1.13 (0.97, 1.33)	0.123	1.40 (1.15–1.70)	0.001	1.04 (0.89–1.21)	0.624
	CVD mortality	1.00 (refer.)	1.14 (0.91, 1.43)	0.261	1.43 (1.13–1.82)	0.004	1.45 (1.22–1.71)	<0.001
	Chronic lower respiratory disease mortality	1.00 (refer.)	1.21 (1.10, 1.34)	<0.001	1.44 (1.29–1.60)	<0.001	1.41 (1.31–1.53)	<0.001
	Alzheimer's disease mortality	1.00 (refer.)	0.67 (0.26, 1.74)	0.408	2.20 (1.09–4.42)	0.027	1.71 (0.91–3.23)	0.098
	Diabetes mellitus mortality	1.00 (refer.)	10.97 (6.28, 19.16)	<0.001	11.04 (6.20–19.64)	<0.001	24.11 (15.55–37.38)	<0.001
	Influenza and pneumonia mortality	1.00 (refer.)	0.97 (0.43, 2.21)	0.95	0.55 (0.20–1.55)	0.258	1.29 (0.76–2.20)	0.342
	Kidney disease mortality	1.00 (refer.)	1.83 (0.84, 3.98)	0.129	4.29 (2.02–9.12)	<0.001	2.25 (1.35–3.73)	0.002
**Breast (women)**								
	All-cause mortality	1.00 (refer.)	1.22 (0.94, 1.56)	0.13	1.13 (0.88–1.46)	0.328	1.38 (1.16–1.64)	<0.001
	Cancer mortality	1.00 (refer.)	0.96 (0.62, 1.47)	0.849	1.26 (0.82–1.94)	0.283	0.89 (0.64–1.26)	0.521
	CVD mortality	1.00 (refer.)	1.18 (0.64, 2.18)	0.588	0.87 (0.41–1.87)	0.727	1.31 (0.87–1.99)	0.2
	Chronic lower respiratory disease mortality	1.00 (refer.)	1.22 (0.95, 1.57)	0.125	1.14 (0.88–1.47)	0.311	1.37 (1.15–1.63)	<0.001
	Alzheimer's disease mortality	1.00 (refer.)	0.16 (0.02, 1.21)	0.076	0.51 (0.06–4.14)	0.526	2.35 (0.73–7.56)	0.153
	Diabetes mellitus mortality	1.00 (refer.)	4.92 (1.42, 16.99)	0.012	5.61 (1.45–21.68)	0.013	13.45 (4.77–37.97)	<0.001
	Influenza and pneumonia mortality	1.00 (refer.)	1.63 (0.35, 7.53)	0.529	1.65 (0.38–7.07)	0.501	0.53 (0.11–2.48)	0.414
	Kidney disease mortality	1.00 (refer.)	0.66 (0.07, 6.62)	0.72	NA	NA	5.41 (1.84–15.87)	0.002
**Prostate**								
	All-cause mortality	1.00 (refer.)	1.11 (0.86, 1.42)	0.424	1.42 (1.07–1.89)	0.015	1.17 (0.96–1.41)	0.118
	Cancer mortality	1.00 (refer.)	1.31 (0.90, 1.91)	0.156	1.28 (0.74–2.20)	0.381	0.92 (0.65–1.31)	0.644
	CVD mortality	1.00 (refer.)	1.05 (0.58, 1.92)	0.871	1.51 (0.81–2.83)	0.194	1.12 (0.74–1.71)	0.592
	Chronic lower respiratory disease mortality	1.00 (refer.)	1.11 (0.86, 1.43)	0.41	1.43 (1.08–1.90)	0.014	1.17 (0.97–1.42)	0.107
	Alzheimer's disease mortality	1.00 (refer.)	0.96 (0.12, 7.74)	0.972	NA	NA	1.89 (0.42–8.56)	0.405
	Diabetes mellitus mortality	1.00 (refer.)	10.18 (2.16, 48.02)	0.003	16.29 (3.29–80.75)	0.001	25.25 (7.85–81.25)	<0.001
	Influenza and pneumonia mortality	1.00 (refer.)	0.21 (0.03, 1.57)	0.127	0.72 (0.09–5.91)	0.757	0.31 (0.05–1.73)	0.179
	Kidney disease mortality	1.00 (refer.)	2.72 (0.47, 15.59)	0.261	5.60 (2.41–13.03)	<0.001	0.60 (0.06–5.83)	0.661
**Colorectal**								
	All-cause mortality	1.00 (refer.)	1.19 (0.90, 1.58)	0.219	1.34 (0.98–1.82)	0.064	1.32 (1.08–1.60)	0.006
	Cancer mortality	1.00 (refer.)	1.29 (0.85, 1.98)	0.236	1.57 (0.91–2.72)	0.106	1.31 (0.90–1.89)	0.154
	CVD mortality	1.00 (refer.)	1.13 (0.55, 2.29)	0.743	0.98 (0.46–2.09)	0.956	1.42 (0.88–2.29)	0.149
	Chronic lower respiratory disease mortality	1.00 (refer.)	1.20 (0.91, 1.59)	0.198	1.34 (0.99–1.83)	0.059	1.33 (1.09–1.62)	0.005
	Alzheimer's disease mortality	1.00 (refer.)	1.21 (0.10, 14.85)	0.881	2.91 (0.54–15.76)	0.215	0.47 (0.06–3.82)	0.476
	Diabetes mellitus mortality	1.00 (refer.)	0.71 (0.08, 6.33)	0.756	5.54 (1.10–27.86)	0.038	23.47 (7.43–74.17)	<0.001
	Influenza and pneumonia mortality	1.00 (refer.)	NA	NA	NA	NA	0.49 (0.08–3.09)	0.444
	Kidney disease mortality	1.00 (refer.)	0.98 (0.10, 9.18)	0.985	0.99 (0.19–5.11)	0.994	0.73 (0.22–2.43)	0.604

*CVD, cardiovascular disease; HR, hazard ratios*.

*Adjusted for age, sex, race, education level, income level, marital status, body mass index, smoking status, alcohol intake, physical activity, cancer diagnosed age, duration of cancer, history of hypertension, coronary heart disease and stroke, and missing data*.

We conducted a sensitivity analysis excluding individuals who were followed up for less than the first 2 years, those with CHD, stroke. Exclusion of individuals with possible type 1 diabetes did not largely affect the HRs indicating the robustness of results (data not shown).

## Discussion

This large prospective study showed that compared to cancer survivors without diabetes, cancer survivors with diabetes had higher risk of all-cause, cancer-specific, and CVD mortality. Further they had an increased risk of all-cause, but not cancer specific mortality as the duration of diabetes increased. These findings add to a largely equivocal evidence base on this relationship.

Diabetes elevates all-cause and cancer specific mortality is consistent with a recent study which showed similar findings in individuals with established prostate cancer (11) as well as other studies in patients with breast and colorectal cancer (8). Some types of cancer are treated less aggressively in cancer survivors with diabetes than those without diabetes (18). In our study, pre-diagnosis diabetes increased the risk of all-cause mortality after adjusting for covariables in breast and prostate cancer sites, which is not consistent with previous findings (19, 20). But additional evidence indicated that pre-existing diabetes increased the risk of all-cause mortality among women with breast cancer after adjusting for covariables related to delayed diagnosis and therapy (21). In addition, pre-diagnosis diabetes increased the risk of all-cause mortality in colorectal cancer survivors in our study, which is consistent with findings in previous study (20).

Our second finding, that all-cause (but not cancer specific) mortality risk increased with diabetes duration has been observed previously in those with colorectal and breast cancer, respectively. With respect to the former group, diabetes duration of more than 10 years was associated with a 49% higher risk of all-cause mortality (22), which is slightly greater than the magnitude of association we observed (33%). With respect to the latter group, we found the risk of all-cause mortality increased in breast cancer patients who have had diabetes for more than 10 years, while others have shown increased risk from 7 years (23).

Among non-diabetic patients, hyperinsulinemia associated with reduced insulin sensitivity may play a role in the pathogenesis of prostate carcinoma (24). Thus, our study might underestimate the effect between insulin secretion or increase of insulin resistance and mortality among cancer survivors. Cancer survivor with diabetes have the same CVD risk of mortality during the second 5 years of diabetes and above 10 years as compared to non-diabetic patients. But this is not aspected since type 2 diabetes abnormalities (such as reduced insulin secretion, increase of insulin resistance) start several years before that the diabetes clinical manifestations appears.

Previous studies indicated the possible relationship between diabetic status and less aggressive oncological treatment and worse overall survival (18). In our study, all-cause mortality (but not cancer-specific) increase in long-standing diabetes diagnosis (>10 years) is likely to be related with the increase of potentially fatal events related to diabetic complications partially explaining the reduced cancer-mortality observed in this group in comparison with the 5–10 years group. A slight increase cancer-related mortality in recently diagnosed diabetic patient (0–5 years group) is likely to be related with short-time exposure to the risk of development of diabetes complications able to influence the oncological treatment strategies.

Diabetes could increase the risk of all-cause mortality among population with different diseases. In our study, both pre- and post-diagnosis diabetes increased the risk of all-cause mortality by 35% than non-diabetic cancer survivors, which was higher than that with 23% among tuberculosis patients (25). Previous studies indicated that patients with diabetes who underwent incident amputation have a 55% higher risk for all-cause mortality than non-diabetic patients (26), and diabetes increased the total mortality markedly with a higher 70% among patients hospitalized with a confirmed acute myocardial infarction than non-diabetic patients (27). In addition, individuals with vs. those without diabetes were at increased higher risk of all-cause mortality in the earlier (144%) and later time (95%) periods in the Framingham Heart Study (28).

There are several mechanistic pathways by which diabetes may confer elevated mortality risk in individuals with cancer history. Cancer patients with diabetes often have other diabetes-related comorbid conditions, which may influence physicians' clinical decision making (29). For example, cancer survivors with diabetes have been shown to be treated less aggressively with chemotherapy and radiotherapy (18, 30). This could partially explain the worse prognosis compared to those without diabetes. Secondly, individuals with co-morbid cancer and diabetes may have poor response to cancer treatment, including increased infection rates and intraoperative mortality (31). Thirdly, they may have a greater risk of chemotherapy-related toxicity (i.e., dilated cardiomyopathy in breast cancer) (30). Thus, conservative clinical decision making or side effects of conventional cancer treatments may impair survival time for individuals with this comorbidity.

The presence of diabetes may itself influence cancer progression via physiologic processes of hyperinsulinemia, hyperglycemia, immunodeficiency, and chronic inflammation. It is possible that most glucose uptake in cancer cells is constitutively high and independent of insulin binding to insulin-like growth factor receptor, which could stimulate cancer cell proliferation and metastasis (32, 33). Another potential pathway is that acute exposure to hyperglycemia may increase endothelial cell permeability for reasons of increased generation of reactive oxidative species and structural changes in the basement membrane, increasing the probability of metastasis (34). The third possible pathway is that hyperinsulinemia related to underlying insulin resistance might stimulate tumor growth (35). Multiple downstream signaling pathways are activated after insulin receptors interact with their ligands, and can stimulate multiple cancer phenotypes contribute to tumor initiation and progression (36). Decreased immunity induced by diabetes can also lead to cancer progression. In this study, the risk of diabetes related mortality increased obviously among cancer survivors with diabetes when compared to non-diabetic survivors. The increased mortality can be reversed when diabetes is managed well in clinical practice.

### Limitations

Our study has several strengths including the large, prospective nature of the cohort used. The representativeness of the sample further adds to the robustness of the findings. Nonetheless, our study had certain limitations. Firstly, we used an epidemiological definition of type 1 diabetes, which implies that misclassification of diabetes type is possible. However, a validation study has shown that those with possible type 1 diabetes adults defined by using insulin and age of onset <30 years old which has been validated as accurate in 97% of cases (16). This could be a potential bias for patients selection in our study. Secondly, we did not have extensive clinical parameters regarding disease status for diabetes and its comorbidities and cancer. For instance, insulin therapy has been associated with increased risk of newly developed colorectal cancer (37), however we could not account for this in the model. Thirdly, lacking of informations about pharmacological and/or surgical treatment received for either diabetes or cancer fail to identify subgroups of cancer survivors who may have been at risk of high mortality rate because of the cancer treatment they received. Fourthly, although diabetes assessments were from self-reports, it is always possible to include the participants who had never received the appropriate diagnostic tests of diabetes, and to misclassify the diabetes as non-diabetes. Self-report of cancer duration or diabetes implies high probability of incorrect data assumed as correct. Finally, our findings may not be generalizable to other cancer sites except for breast, colorectal, and prostate cancer.

## Conclusion

Breast, prostate, and colorectal cancer survivors with diabetes had higher risk of all-cause mortality than did cancer survivors without diabetes. Pre-diagnosis diabetes increased risk of all-cause mortality after adjusting for covariables in breast and prostate cancer sites. Duration of diabetes is associated with increased risk of all-cause but not cancer specific mortality when compared to non-diabetic breast and prostate cancer survivors. Greater attention on diabetes control might be warranted in those with comorbid cancer and diabetes.

## Data Availability Statement

The datasets generated for this study are available on request to the corresponding author Yafeng Wang (wonyhfon@163.com).

## Ethics Statement

The studies involving human participants were reviewed and approved by all procedures performed in studies involving adult participant provided a written informed consent and the NHIS was approved by the National Center for Health Statistics ethics review board. The patients/participants provided their written informed consent to participate in this study. Written informed consent was obtained from the individual(s) for the publication of any potentially identifiable images or data included in this article.

## Author Contributions

YW, XC, and YJ: conception and design of the study. YW and HT: acquisition of data and analysis, and statistical analysis. HT, YW, AO'N, YC, WW, JW, XC, and YJ: writing and revision of the manuscript. All authors read and approved the final manuscript.

### Conflict of Interest

The authors declare that the research was conducted in the absence of any commercial or financial relationships that could be construed as a potential conflict of interest.

## Abbreviations

NHIS: National Health Interview Survey
DM: Diabetes mellitus
CVD: Cardiovascular disease
CHD: Coronary heart disease
BMI: Body mass index
MET: Metabolic equivalent task
HRs: Hazard ratios
CI: Confidence intervals.
